# Comparative analysis of host responses to Oropouche viruses in placental cell infection models

**DOI:** 10.1371/journal.pntd.0014593

**Published:** 2026-09-21

**Authors:** Katie A. Latham, Georgia Brassington, Alan M. Pittman, Isabelle Dietrich, Joseph Hughes, Yvette Kazungu, Jessica A. Plante, Kenneth S. Plante, Andrew Sharp, Benjamin Brennan, Beth Holder, Blair L. Strang, Alain Kohl

**Affiliations:** 1 Department of Medicine, Institute of Infection and Immunity, St George’s School of Health and Medical Sciences, City St George’s, University of London, London, United Kingdom; 2 Liverpool School of Tropical Medicine, Centre for Neglected Tropical Diseases, Departments of Tropical Disease Biology and Vector Biology, Liverpool, United Kingdom; 3 Department of Molecular and Biomedical Sciences, Cardiovascular and Genomics Research Institute, St George’s School of Health and Medical Sciences, City St George’s, University of London, London, United Kingdom; 4 The Pirbright Institute, Ash Road, Pirbright (Woking), United Kingdom; 5 MRC-University of Glasgow Centre for Virus Research, Glasgow, United Kingdom; 6 The Department of Microbiology and Immunology, University of Texas Medical Branch, Galveston, Texas, United States of America; 7 World Reference Center for Emerging Viruses and Arboviruses, University of Texas Medical Branch, Galveston, Texas, United States of America; 8 Harris Research Centre, Department of Women’s and Children’s Health, University of Liverpool, United Kingdom; 9 Liverpool Women’s Hospital NHS Foundation Trust, Liverpool, United Kingdom; 10 Institute of Reproductive and Developmental Biology, Department of Metabolism, Digestion, and Reproduction, Imperial College London, London, United Kingdom; Air Force Medical University, CHINA

## Abstract

2022-2024 saw a major outbreak of midge-borne Oropouche virus (OROV, *Peribunyaviridae*) that affected Brazil and countries across the region and was associated with a novel reassortant of this pathogen. The description of fetal deaths, microcephaly and congenital malformations in newborns led to the question whether such pathologies had previously been overlooked or are associated with novel properties of reassortant OROV, raising the question of infection and transfer across the placenta. Here we compared infection of cultured placental cell lines (BeWo and JEG-3) with an earlier OROV isolate and a Cuban isolate from 2024. Our data suggest minor, but noticeable differences between these OROV and respective host cell responses, for example relating to IFNL3. It remains to be investigated whether characteristics of these different isolates translate to effects on fetal development, but these findings suggest that cellular responses can differ between ancestral and recent isolates of this virus.

## Author summary

Oropouche virus (OROV) is a midge-borne human pathogenic orthobunyavirus that caused an unexpected, large outbreak around 2022–2024 in Brazil and other parts of the region. This was linked to a novel reassortant, e.g., a virus whose genome is a new combination of the three negative strand RNA segments that make up the viral genome. During this outbreak, OROV infection was associated with occasional cases of fetal death, microcephaly and congenital malformations in newborns which had not been described before. This raised the question whether this reassortant OROV had properties different from other OROV, including infection of the placenta and from there the fetus. Here we compared an ancestral and a recent OROV to assess their ability to infect placental cell and induction host responses. While differences were generally minor, some noticeable changes may be relevant to understand the pathogenesis of this infection.

## Introduction

Oropouche virus (OROV) is an arbovirus that is mainly transmitted by *Culicoides* midges, with *Culicoides paraensis* as primary vector for human infections during urban epidemic outbreaks [1]. Traditionally associated with febrile disease and myalgia (with rare neurological complications), the 2022–2024 OROV outbreak was characterised by transmission in non-endemic regions of Brazil and beyond, leading to large numbers of human infections [2–5], and deaths in adults [6,7]. A previously undescribed, potential association was made between OROV and fetal deaths, congenital malformations and microcephaly [8–12]. Additionally, re-examination of historical cases of microcephaly with unknown cause in these locations suggests a potential link with OROV infection [8–13]. Together, these data suggest vertical transmission from mother to fetus across the placenta, impacting on embryonal/fetal development. This raises the prospect of new public health impacts by OROV, particularly outside endemic areas [14], in a manner reminiscent of experiences with Zika virus (ZIKV, *Flaviviridae*) in the Americas [15]. A number of questions on the tropism and pathology associated with OROV in pregnancy remain open, despite this new association between maternal infection and severe fetal outcomes.

OROV is a pathogen of the *Peribunyaviridae* family, *Orthobunyavirus* genus. The peribunyaviral genome consists of three segments of single-stranded, negative-strand RNA called L (encoding the viral RNA dependent RNA polymerase), M (encoding the structural glycoproteins Gn and Gc, as well as the NSm protein), and S (encoding the nucleocapsid protein N and a type I interferon antagonist called NSs). Complementary segment termini result in viral ribonucleoproteins forming so-called panhandle structures. Replication is cytoplasmic, and the segmented nature of the genome allows evolution through reassortment, should co-infection of viruses of two (or more) the same or closely related species arise. Reassortment has been shown to result in new virus properties, for example, alterations in tropism and/or replicative properties [2,16–22].

The 2022–2024 OROV outbreak was associated with the appearance of a novel reassortant lineage of OROV, with cases imported to North America and Europe from the Amazon, and possessing an M segment derived from OROV found in the Amazon and S and L segments of OROV found in Colombia, Peru and Ecuador [23–26]. This novel OROV reassortant could be key to why OROV appears to be newly associated with reported pregnancy loss and birth defects. However, association of previously circulating OROV lineages with congenital infection has not been rigorously investigated, making it difficult to come to clear conclusions as to whether novel OROV has a specific role in human disease. Indeed, the association may just have been overlooked and previously determined OROV exposure in historical microcephaly cases suggests that vertical transmission of OROV has occurred in the past [11]. A recent study has shown that a Brazilian prototype strain of OROV isolated in 1960 (strain BeAn19991) is capable of infecting placental trophoblasts as well as placental explants, presenting a route for vertical transmission [27] while both prototype (BeAn19991) and recent (2400023, isolated in 2024 from infection in Cuba) OROV isolates can infect placental cell lines such as BeWo [28]. Nonetheless, it remains unclear how recent OROV compares to previously circulating virus in terms of vertical transmission and comparisons of OROV lineages are a matter of urgency. Indeed, higher replication, earlier plaque formation and sizes and reduced neutralising capacity from historical sera observed with reassortant OROV indicate considerable shifts in properties of OROV over time [26]. Here, we used placental cell lines to study the infection properties of an ancestral lineage in comparison to a recent isolate and determine differences between these viruses.

## Methods

### Cells and viruses

BeWo and JEG-3 cells were obtained from the American Type Culture Collection no. CCL-98 & no. HTB-36 respectively (ATCC). A549-IRF3-KO cells lacking expression of the innate immune activator IRF3, were obtained from Abcam (ab267098). BeWo cells were maintained in Dulbecco’s Modified Eagle media-F12 Nutrient Mixture (DMEM/F-12 with 2.5mM L-glutamine and 15mM HEPES; Gibco, 11330–032), JEG-3 cells were maintained in Minimal Essential Media (with Earle’s salts and L-Glutamine; Gibco, 11095–080). A549-IRF3-KO were cultured in Dulbecco’s Modified Eagle media (DMEM with 4.5g/L D-Glucose, L-Glutamine and pyruvate; Gibco, 41966–029). All cell culture media also contained 10% fetal bovine serum (FBS) (Gibco, A5256801) and 1% penicillin-streptomycin (Gibco, 15070–063). Cells were cultured at 37°C with 5% CO_2_. An ancestral OROV strain (NCPV 1409261v, from NCPV records isolated in 2010) was obtained from UKHSA Culture Collections, and in this study was referred to as OROV1_2010. A recently isolated Cuban OROV strain (UTMB 2400023) was obtained from the World Reference Centre for Emerging Viruses and Arboviruses at University of Texas Medical Branch (in this study is referred to as OROV2_2024). Stocks of both OROV strains were generated by low multiplicity of infection (MOI) of A549-IRF3-KO cells and collection of cell supernatant 96 hours post infection (hpi). Virus was stored at -80°C. Viral sequences were verified as indicated below.

### Determination of viral titre

OROV titres were determined by serial dilution of OROV supernatant onto BeWo, JEG-3 or A549-IRF3-KO monolayers (1x10^5^ cells per well of a 24-well plate), which were then overlayed with their respective media containing 5% FBS and 0.4% agarose. After 72 hours incubation at 37°C with 5% CO_2_, cells were fixed using 4% paraformaldehyde in phosphate buffered saline (PBS) (pH 7.2) and stained with 1% crystal violet in ddH_2_O. Plaques in the infected cell monolayers were counted and OROV titre was expressed as plaque forming units per millilitre/mL (pfu/ml). Plaque diameters were measured using ImageJ Version 2.0.

### Flow cytometry

BeWo and JEG-3 cells (5x10^5^ cells per well of a 6-well plate) were infected with OROV1_2010 or OROV2_2024, using conditions outlined in the text and figure legends. After 1 hour, viral supernatant was removed and fresh media added. At 18 hours post infection, infected cells and uninfected control cells were trypsinized, washed and fixed in 4% paraformaldehyde in phosphate buffered saline (PBS) (pH 7.2) for 1 hour at room temperature. Cells were then aliquoted at ~150,000 cells per well onto a round bottom 96 well plate in Flow Cytometry buffer (PBS containing 1% Bovine Serum Albumin (BSA) (Capricorn Scientific, CP2–7044) and 0.01% sodium azide). Cells were pelleted for 5 minutes at 400x*g* and resuspended in OROV virus immune murine ascitic fluid (V-505–701 562, ATCC) diluted 1:1000 in a solution of 0.1% TritonX-100 (Thermo Scientific A16046.Ap) in PBS and incubated at 4°C for 30 minutes. Cells were washed with Flow Cytometry buffer, centrifuged for 5 minutes at 400x*g* and incubated with 1µg/ml goat anti-mouse Alexa Fluor 488 secondary antibody (Invitrogen, A28175) at 4°C for 20 minutes. Cells were washed twice and resuspended in Flow Cytometry buffer prior to analysis using Cytek Aurora cytometer. For all experiments, at least 10,000 live events were obtained and gated to singlets. Data were analysed using FlowJo V10 to determine % positive antibody-stained cells.

### RNA sequencing

For transcriptome analysis, BeWo and JEG-3 cells (5x10^5^ cells per well of a 6-well plate) were infected with OROV1_2010 or OROV2_2024 at a MOI of 0.1 or 1.0 for 1 hour at 37°C with 5% CO_2_. After incubation, virus media was removed and fresh respective cell media with 10% FBS and 1% P/S were added. After 18 hours, cell culture supernatant was removed, and cells were lysed using TRIzol (Invitrogen), according to the manufacturer’s instructions, and lysate was stored at -80°C. TRIzol lysates were sent to Genewiz (Azenta Life Sciences) for RNA purification, preparation of sequencing libraries and sequencing. mRNAs were enriched using Oligo(dT) beads, and RNA sequencing libraries were prepared using a NEBNext Ultra II RNA Library Kit for Illumina (NEB). The sequencing libraries were then validated on an Agilent Fragment Analyzer (Agilent Technologies) followed by quantification on a Qubit Fluorometer (Invitrogen). Libraries were sequenced to generate 150-bp paired-end reads using an Illumina sequencing platform. The raw sequencing data (bcl files) generated were converted into fastq files and de-multiplexed with Illumina bcl2fastq software. Sequences were then aligned to the human reference genome (hg38) with STAR (version 2.6.8a) [29]. Differential gene expression was performed using DESeq2 (v1.30.1) [30] in R (v3.8.3). Differentially expressed genes (DEGs) were defined as cellular genes with log_2_ (fold-change) >1 and adjusted P value <0.05. Gene Ontology (GO) analysis was carried out with DAVID (National Institutes of Health, USA) [31,32]. Raw sequence reads were deposited as outlined.

For viral RNA sequencing, total RNA was isolated from 2.5x10^5^ A549-IRF3 KO cells infected at MOI of 1 for 24 hours, and TRIzol lysates were sent to Genewiz (Azenta Life Sciences) for RNA purification. rRNA was depleted using a FastSelect rRNA HMR kit (Qiagen), and RNA sequencing libraries prepared using a NEBNext Ultra RNA Library Kit for Illumina (NEB). The sequencing libraries were then validated using NGS Kit on an Agilent High Sensitivity D1000 ScreenTape (Agilent Technologies) and following this quantified using a Qubit 4.0 Fluorometer (Invitrogen). Libraries were sequenced on an Illumina NovaSeq X using a 2x150 pair-end configuration. Again, raw sequencing data (bcl files) generated were converted into fastq files and de-multiplexed with Illumina bcl2fastq software. Raw sequence reads were deposited as indicated. For reconstruction of OROV genomes, raw Illumina reads were adapter- and quality-trimmed using Trim Galore (v0.6.6) and aligned to the corresponding GenBank reference sequences (L: NC_005776.1; M: NC_005775.1; S: NC_005777.1) using BWA (v 0.7.1 7) [33]. Consensus sequences were generated with SAMtools (v1.12) [34] using a minimum depth of 10 reads, retaining insertions and deletions (--show-ins yes --show-del yes).

### Phylogenetic analysis

The two OROV newly generated genomes were added to a curated dataset from Nextstrain (updated 2026-01-01). Segment-specific multiple sequence alignments were produced with MAFFT (v7.475) [35]. Maximum-likelihood phylogenies were inferred using IQ-TREE (v3.0.1) [36], with substitution models selected by BIC and node support estimated from 1,000 bootstrap replicates.

### Virus replication assay

BeWo cells were seeded (2.5x10^4^ cells per well of 24-well plate) 72 hours prior to infection. At 48 hours prior to infection, cells were syncytialised by addition of 20μM forskolin or 20μM DMSO as carrier control. BeWo cells were then infected with OROV1_2010 or OROV2_2024 at a MOI of 0.1 or 1 for 1hr at 37°C with 5% CO_2_. After infection, viral supernatant was removed and DMEM-F12 with 10% FBS and 1% P/S was added for 18 hours at 37°C with 5% CO_2_. Supernatants were collected and stored at -80°C. Viral replication was analysed using a plaque assay (outlined above) to determine viral titre.

### Statistical analysis

All statistical testing and graphing were performed using GraphPad Prism software (version 10.2.3). Two-tailed unpaired t-tests were performed to compare viral titre between strains and experimental conditions. A one-way ANOVA test was used to compare strains and conditions for flow cytometry. Differences were considered significant when p values were <0.05.

## Results

### Infection of placental cell lines by OROV1_2010 and OROV2_2024

Here we sought to compare infection levels in placental cell lines of a previously isolated Brazilian strain of OROV (OROV1_2010) and a recent Cuban OROV strain (OROV2_2024).

**Fig 1:**
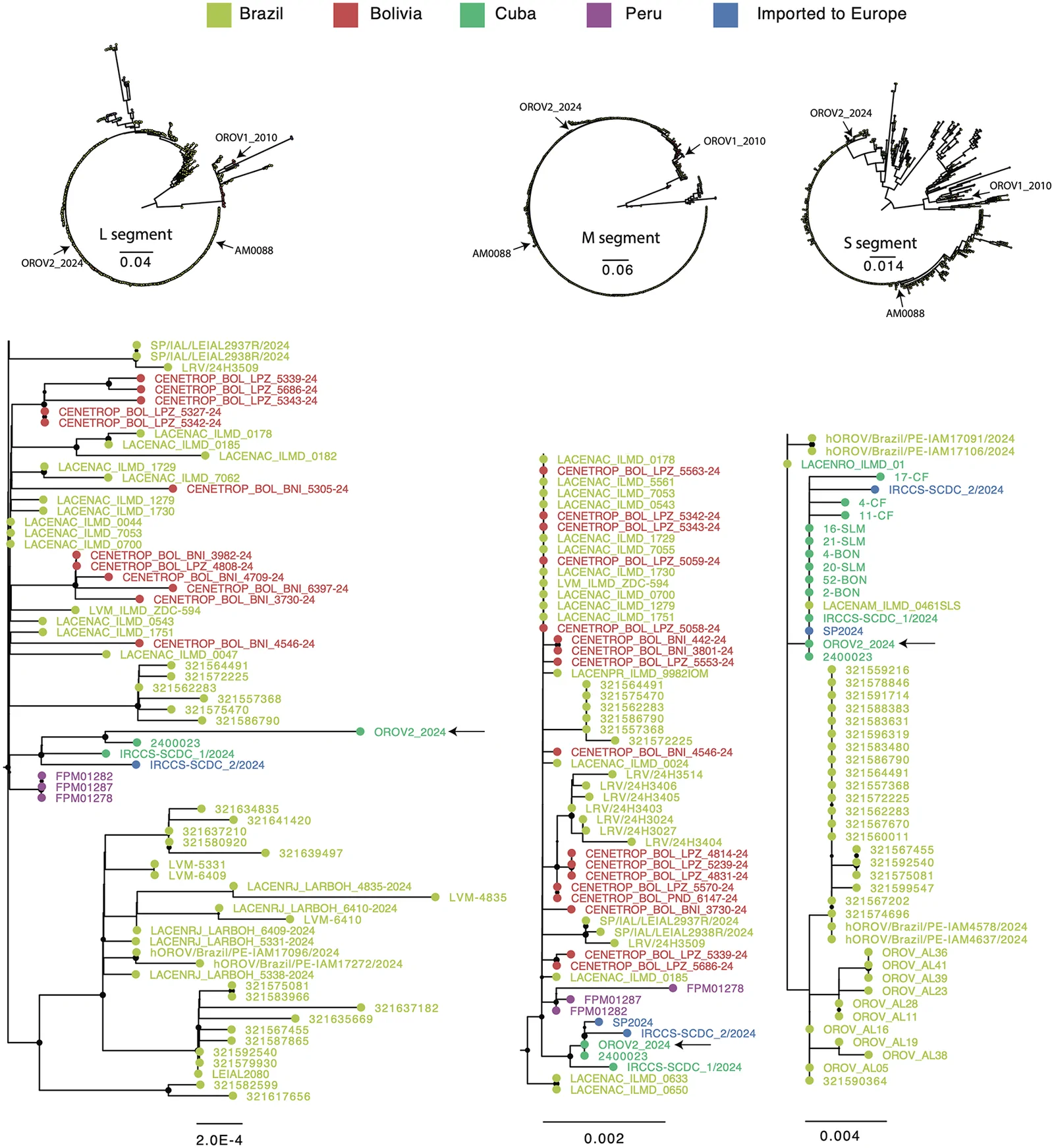
Phylogenetic analysis of OROV1_2010 and OROV2_2024 genomes. Maximum-likelihood phylogenies for each genomic segment shown as circular trees, reconstructed using all publicly available sequences from GenBank (dataset curated via Nextstrain). The subtree containing OROV2_2024 is expanded below, with tip colours indicating country of origin. Nodes with bootstrap support ≥90% are marked with black circles.

To confirm the genomic integrity of OROV used here, viral genomic RNA was sequenced from infections of A549-IRF3-KO cells. Phylogenetic analyses indicate that the 2024 Cuban isolate (OROV2_2024) clusters within a clade comprising recent OROV sequences, including other contemporary isolates from Cuba. Across segments, the 2024 genome is more closely related to the recent outbreak associated isolate AM0088 [26] than to the earlier isolate (OROV1_2010). However, phylogenetic resolution varies by segment: the L segment provides the strongest support for clustering with recent Cuban sequences, whereas the M and S segments show lower resolution and broader regional mixing, limiting confidence in precise geographic origin (Fig 1). Overall, the data support the interpretation that the 2024 Cuban isolate represents a recent lineage that clusters with contemporary outbreak-associated sequences, related to recent OROV infections potentially linked to vertical transmission, resulting in pregnancy loss and birth defects in humans [37]. The sequence data provided here provide a genetic baseline for the viruses as used during this project. This may be useful for future research using these or related viruses.

**Fig 2:**
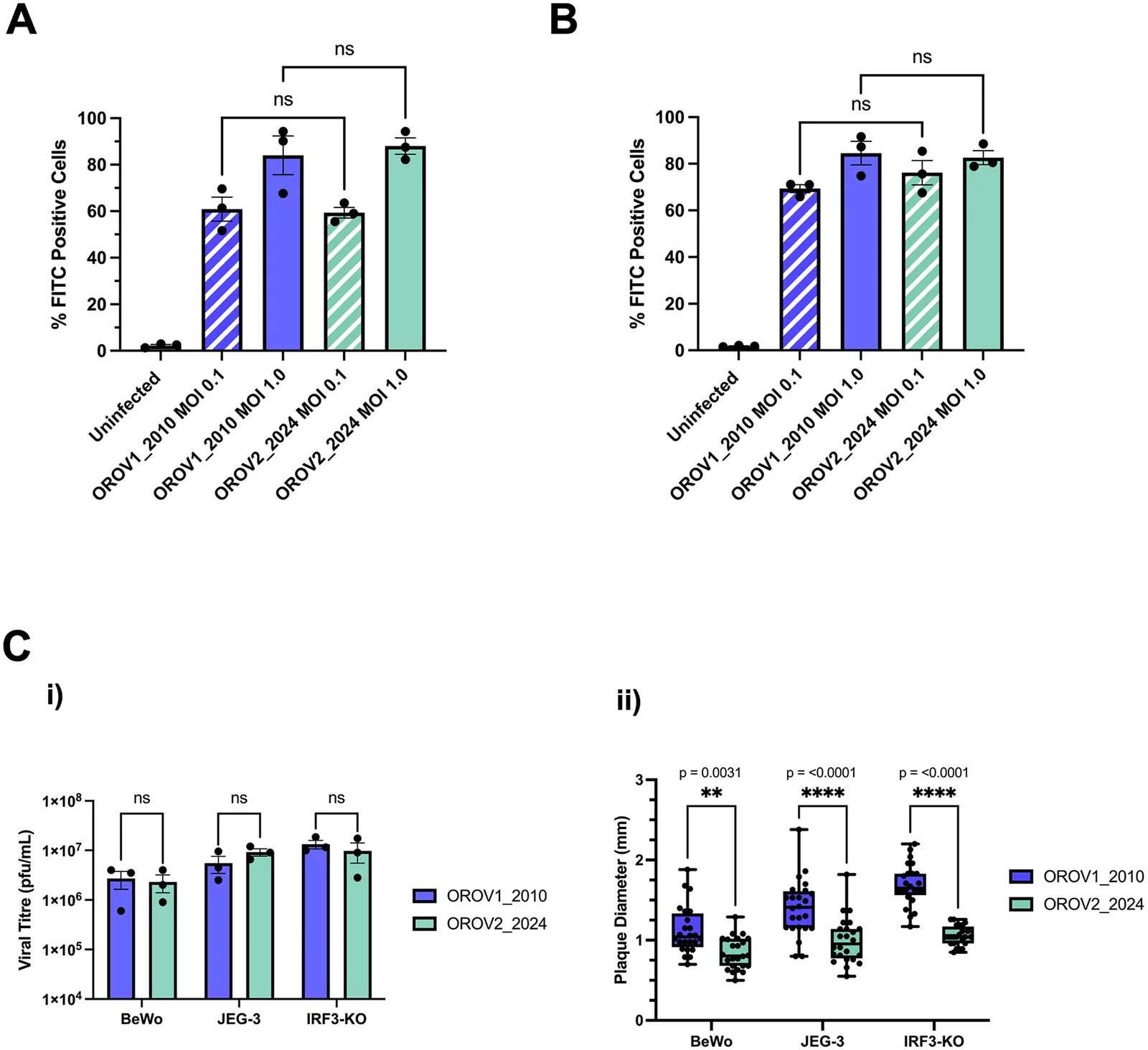
Characteristics of OROV1_2010 and OROV2_2024 infection of placental cells. BeWo cells **(A)** or JEG-3 cells **(B)** were infected with OROV1_2010 (purple) or OROV2_2024 (green) at MOI 0.1 or MOI 1.0 or uninfected (grey), and percentage antibody positive cells were evaluated at 18 hpi using flow cytometry. **(C)** BeWo, JEG-3 or A549-IRF3-KO cells were infected with MOI 0.001 of either of OROV1_2010 or OROV2_2024. At 72 hpi, infected cells were stained, **(i)** viral titre and **(ii)** plaques sizes were measured. Plaque sizes were determined using Image J software. **(A, B & C (i))** Blocks and error bars represent the mean and standard error of mean, respectively, of data from three independent experiments. **(C (ii))** Data points represent measurements of eight individual plaques chosen at random from 3 independent experiments, and error bars represent the range. Data was analysed using a one-way **(A & B)** and two-way **(C)** ANOVA; p-values <0.05 (*), < 0.01 (**), < 0.001 (***) or <0.0001 (****) were considered statistically significant.

We then assayed infection of OROV viruses at different MOIs in BeWo cells, a commonly used human choriocarcinoma-derived cell line displaying features similar to cytotrophoblast cells [38]. Using murine ascitic fluid recognizing OROV proteins, we calculated the percentage of infected cells by flow cytometry, measuring the number of antibody-positive cells within uninfected and infected cells (Fig 2A). The number of infected cells increased with an increasing MOI in cells infected with either strain (Fig 2A); at MOI of 0.1 both strains could infect over 50% of the cell population within the first 18 hpi, and this increased to above 80% of the cell population at an MOI of 1. Therefore, both OROV1_2010 and OROV2_2024 successfully infected BeWo cells to comparable levels. We also assayed infection in another commonly used human choriocarcinoma-derived placental cell line, JEG-3. Like BeWo cells, robust infection was observed at both MOIs with both strains (Fig 2B). Therefore, there was no obvious difference in the ability of different OROV strains to enter different trophoblast cell lines.

To assess if this similar ability of OROV strains to infect trophoblast cells also meant similar production of infectious virus we infected BeWo, JEG-3 or A549-IRF3-KO cells with OROV1_2010 and OROV2_2024. Plaque assays were performed to determine viral titre from infected supernatants and measure plaque sizes (Fig 2C). We found no significant difference in viral titre between OROV1_2010 and OROV2_2024 in any of the three cell lines (Fig 2C, 2i). However, there was a difference in plaques sizes, whereby regardless of cell line OROV2_2024 had significantly smaller plaques than OROV1_2010 (Fig 2C, 2ii).

Overall, this suggested that although production of infectious virus did not vary between cell lines, OROV2_2024 had reduced ability to form plaques compared to OROV1_2010 in a plaque assay, which could be linked to replication, cell-to-cell spread, viral release or a combination of processes. To determine the exact mechanism(s) would require further investigations.

### Sequencing the transcriptome from OROV infected cells to assess cellular gene expression patterns

**Fig 3:**
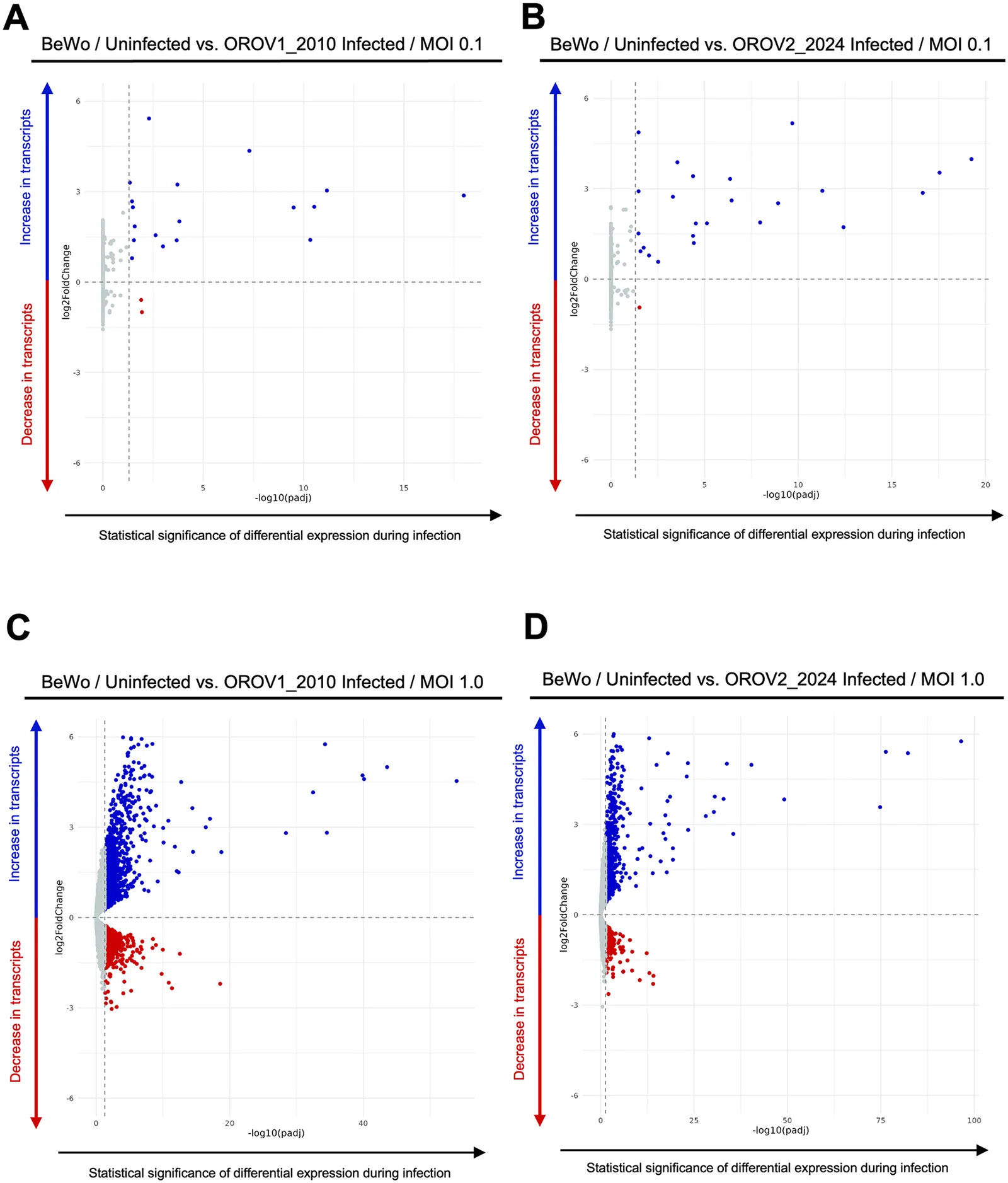
Differential gene expression comparing RNA transcripts in uninfected and infected BeWo cells. BeWo cells were infected at MOI 0.1 **(A, B)** or 1.0 **(C, D)** with either OROV1_2010 **(A and C)** or OROV2_2024 **(B and D)** followed by mRNA RNAseq at 18 hpi (n = 3). Volcano plots show results of differential gene analysis: (A) MOI 0.1 OROV1_2010, (B) MOI 0.1 OROV2_2024, (C) MOI 1.0 OROV1_2010, (D) MOI 1.0 OROV2_2024; with a statistically significant cut off at adjusted P value <0.05. Transcripts upregulated in OROV-infected cells are indicated in blue and downregulated in red.

To further understand potential differences in virus-cell interactions and subsequent host responses, mRNA sequencing was performed on BeWo cells infected with OROV1_2010 or OROV2_2024 and compared to uninfected BeWo cells. The differentially expressed genes (DEGs) are listed in S1 Table. Using a cut off of adjusted P value <0.05 to be considered statistically significant, in BeWo cells infected at MOI 0.1, OROV1_2010 infection resulted in upregulation of 12 transcripts, and OROV2_2024 16 transcripts, neither infection had any downregulated transcripts that met the cutoff of -2 (Fig 3A-3B). At the higher MOI of 1.0, OROV infection resulted in a much larger number of DEGs. OROV1_2010 induced upregulation of 372 genes and downregulation of 16, and OROV2_2024 induced upregulation of 323 genes and downregulation of 5 (Fig 3C-3D, S2 Table).

**Fig 4:**
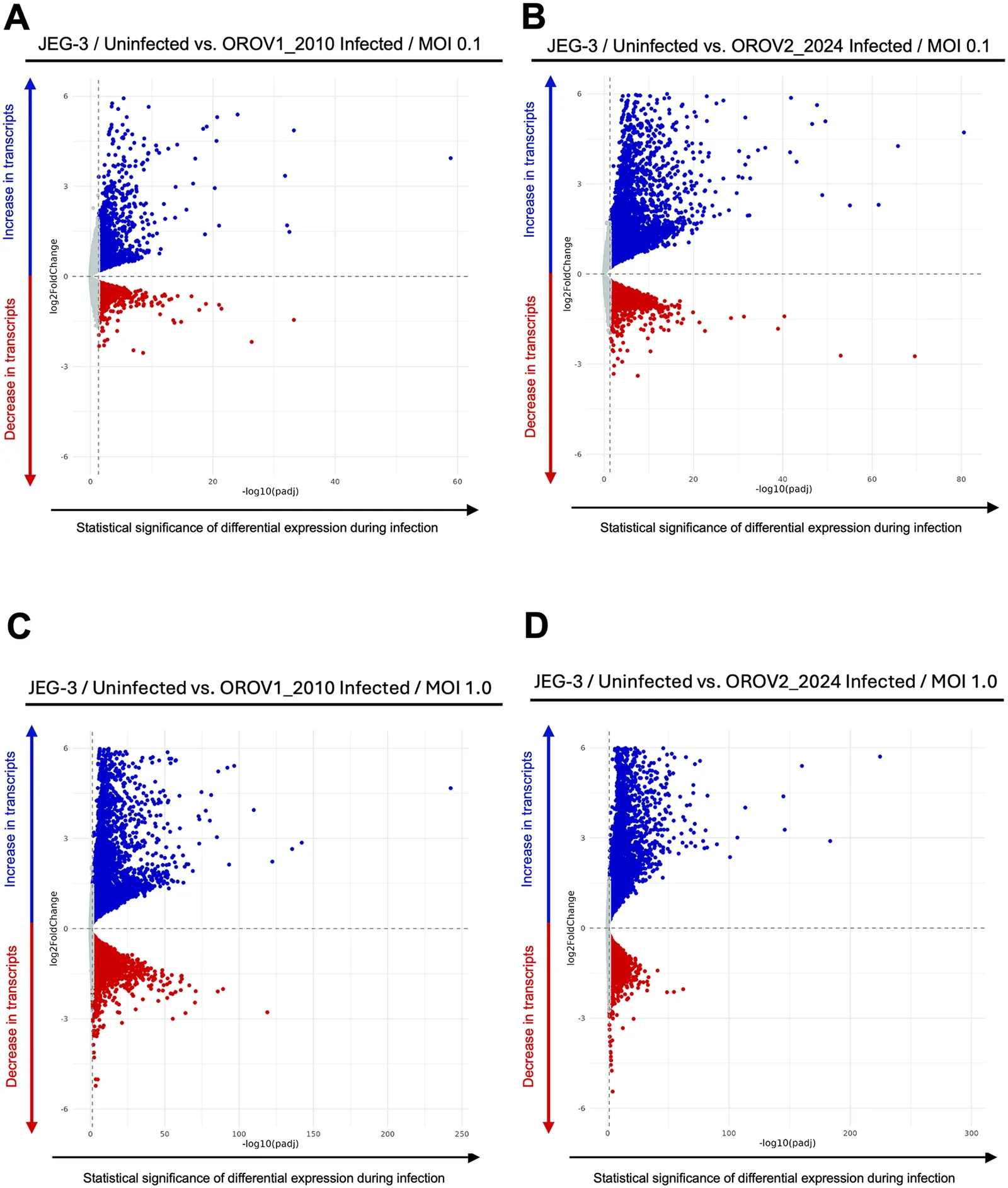
Differential gene expression comparing RNA transcripts in uninfected and infected JEG-3 cells. JEG-3 cells were infected at MOI 0.1 **(A, B)** or 1.0 **(C, D)** with either OROV1_2010 **(A, C)** or OROV2_2024 **(B, D)** followed by mRNA sequencing at 18 hpi. Volcano plots show results of differential gene analysis: (A) MOI 0.1 OROV1_2010, (B) MOI 0.1 OROV2_2024, (C) MOI 1.0 OROV1_2010, (D) MOI 1.0 OROV2_2024; with a statistically significant cut off at adjusted P value <0.05. Transcripts upregulated in OROV-infected cells are indicated in blue and downregulated in red (n = 3).

We also performed mRNA sequencing on JEG-3 infected with OROV1_2010 or OROV2_2024 and compared to uninfected JEG-3 cells. DEGs are listed in S3 Table. In contrast to BeWo cells, in JEG-3 cells infected at MOI 0.1, we found OROV1_2010 induced upregulation of 239 transcripts and downregulation of 7 transcripts, and OROV2_2024 induced upregulation of 1117 transcripts and downregulation of 20 transcripts (Fig 4A-4B). At the higher MOI of 1.0, OROV1_2010 induced upregulation of 1479 transcripts and downregulation of 173 transcripts, and OROV2_2024 induced upregulation of 1992 transcripts and downregulation of 99 transcripts (Fig 4C-4D, S4 Table). Overall, this suggests that there is an MOI- and cell- dependent effect in both up- and down- regulation of RNA transcripts in response to infection.

**Fig 5:**
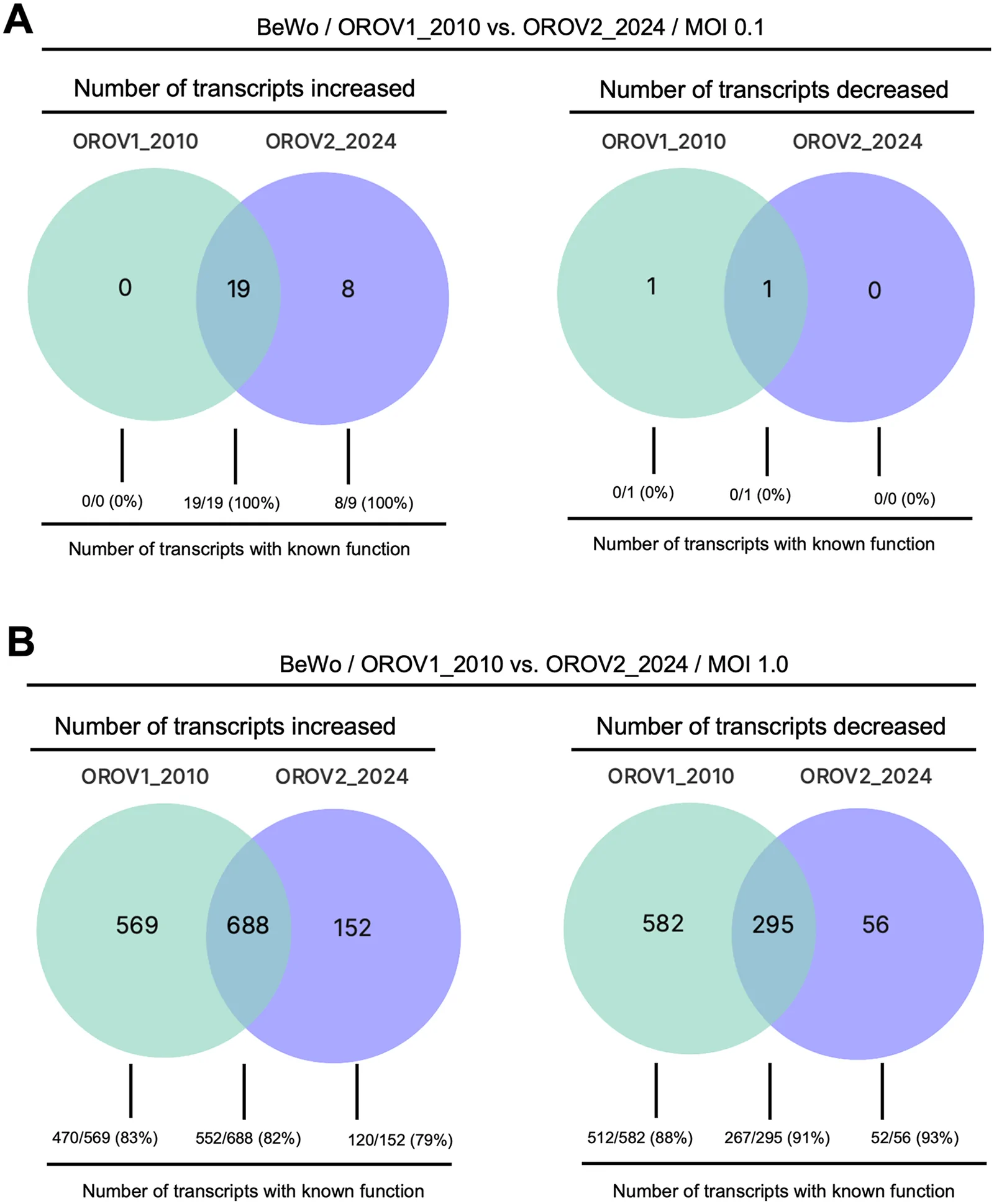
Number of transcripts common or unique to OROV infection at different MOI in BeWo cells. Venn diagrams showing the number of transcripts common or unique to either OROV1_2010 (green) or OROV2_2024 (purple) infection that increased or decreased at either (A) MOI 0.1 or (B) MOI 1.0. Also shown below each figure is the number and percentage of those transcripts with known biological function, determined using DAVID. (https://davidbioinformatics.nih.gov/).

Common or unique DEGs to OROV1_2010 and OROV2_2024 infection in both cell lines and at both MOIs are listed in S5 Table and S6 Table, respectively. In BeWo cells at MOI 0.1 infection, the 19 upregulated DEGs were common between strains and 8 were unique to OROV2_2024 and the downregulated DEGs 1 was common and one unique to OROV1_2010 (Fig 5A). At the higher MOI 1.0 infection, for upregulated DEGS there were 688 common to both strains, 569 unique to OROV1_2010, 152 unique to OROV2_2024, and for downregulated DEGs there were 295 common to both strains, 582 unique to OROV1_2010 and 56 unique to OROV2_2024 (Fig 5B). Thus, at the higher MOI, the two strains elicited more discordant than common DEGs, particularly for downregulated transcripts.

**Fig 6:**
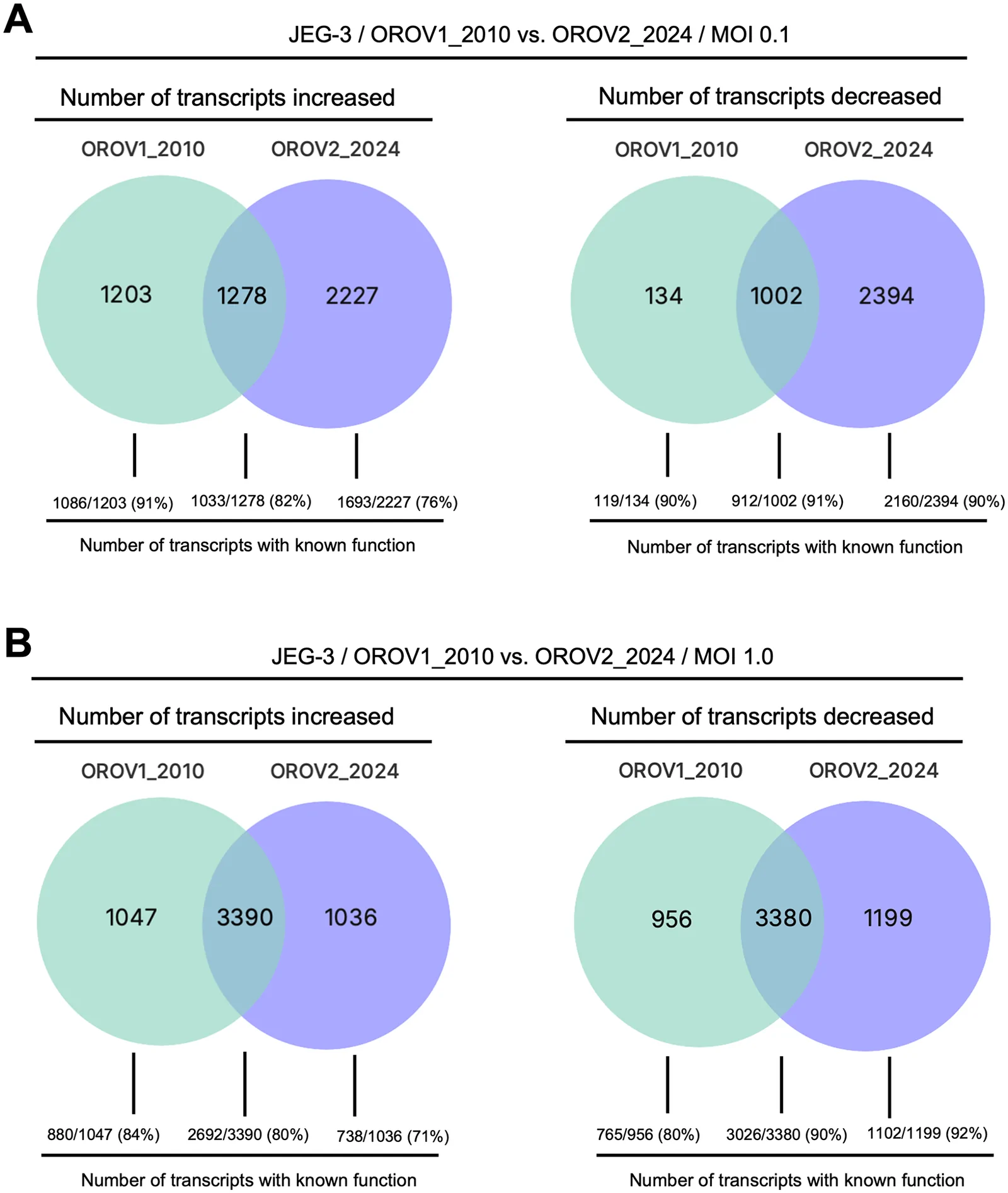
Number of transcripts common or unique to OROV infection at different MOI in JEG-3 cells. Venn diagrams showing the number of transcripts common or unique to either OROV1_2010 (green) or OROV2_2024 (purple) infection that increased or decreased at either (A) MOI 0.1 or (B) MOI 1.0. Also shown below each figure is the number and percentage of those transcripts with known biological function, determined using DAVID (https://davidbioinformatics.nih.gov/).

In JEG-3 infected cells at MOI 0.1 we saw a similar result to that of BeWo cells but with greater transcript numbers (Fig 6A). At MOI 1.0, the upregulated DEGs had 3390 in common, 1047 unique to OROV1_2010, 1036 unique to OROV2_2024 and downregulated DEGs showed 3380 in common, 956 unique to OROV1_2010 and 1199 unique to OROV2_2024 (Fig 6B). This suggests that in JEG-3, conversely to BeWo cells, there are more DEGs both up- and down- regulated in common between the strains than different.

### Functional analysis of RNA transcripts that increased or decreased during OROV infection

**Fig 7:**
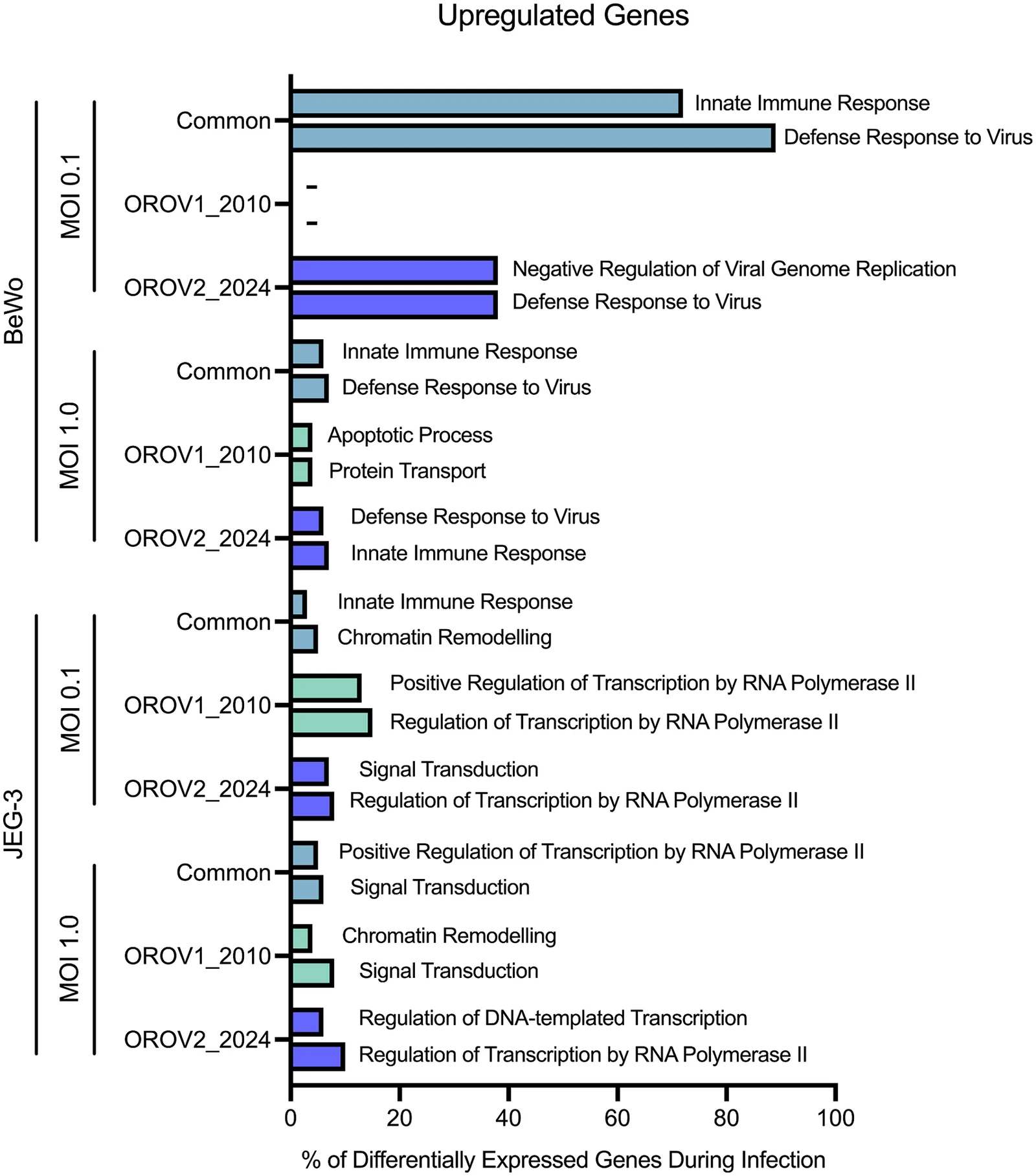
Functional analysis of transcripts that increased during OROV1_2010 or OROV2_2024 infection in both BeWo and JEG-3 cells. Functional analysis of transcripts was carried out using DAVID and the percentage of transcripts assigned to a functional group was recorded in transcripts common or unique to either OROV1_2010 or OROV2_2024 infection that increased in either BeWo or JEG-3 cells. In each condition, the two categories with the greatest percentage of transcripts are shown.

**Fig 8:**
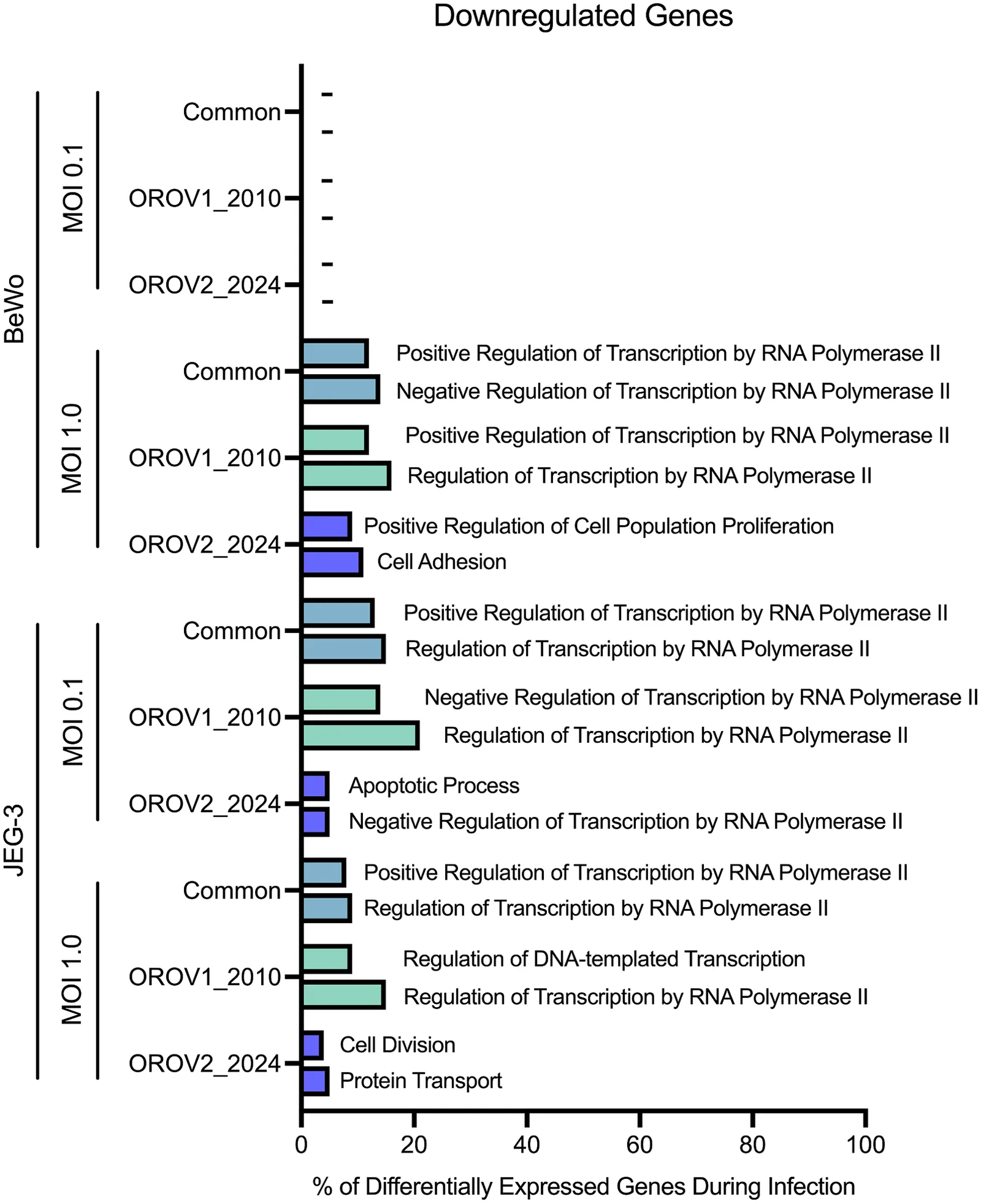
Functional analysis of transcripts that decreased during OROV1_2010 or OROV2_2024 infection in both BeWo and JEG-3 cells. Functional analysis of transcripts was carried out using DAVID analysis and the percentage of transcripts assigned to a functional group was recorded in transcripts common (blue) or unique to either OROV1_2010 (green) or OROV2_2024 (purple) infection that decreased in either BeWo or JEG-3 cells. In each condition, the two categories with the greatest percentage of transcripts are shown.

To identify the function(s) of the DEGs in infected cells (Figs 3 and 4), we used DAVID to analyze gene ontologies. Overall, nearly all of the transcripts identified were associated with a known biological function (Figs 5 and 6). To explore the function of the commonly and discordantly changed transcripts induced by the two strains, we used pathway analysis on the increased (Fig 7) and decreased (Fig 8) DEGs (also see S7 Table and S8 Table). Ranking by percentage of DEGs mapping to each pathway, at MOI 1.0, common DEGs in BeWo cells were most associated with innate immune response and defence response to virus. The unique DEGs induced by OROV2_2024 also associated with these two pathways, but for OROV1_2010 the pathways with the largest percentage of DEGs were apoptotic process and protein transport (Fig 7). In JEG-3 infected cells at MOI 1.0, the common DEGs were associated with regulation of transcription by RNA polymerase II and signal transduction. The unique DEGs for OROV1_2010 also associated with signal transduction but additionally chromatin remodeling, and for OROV2_2024 the largest percentage of DEGs were associated to pathways of regulation of DNA-templated transcription and transcription by RNA polymerase II as seen in the common DEGs (Fig 7). In the decreased transcripts at MOI 1.0 both BeWo and JEG-3 infected cells had DEGs common and unique to OROV1_2010 in pathways associated with regulation of transcription by RNA polymerase II (Fig 8). Whereas the DEGs unique to OROV2_2024 were associated with cellular process such as cell adhesion and regulation of cell population proliferation in BeWo cells, and in cell division and protein transport in JEG-3 cells (Fig 8).

**Fig 9:**
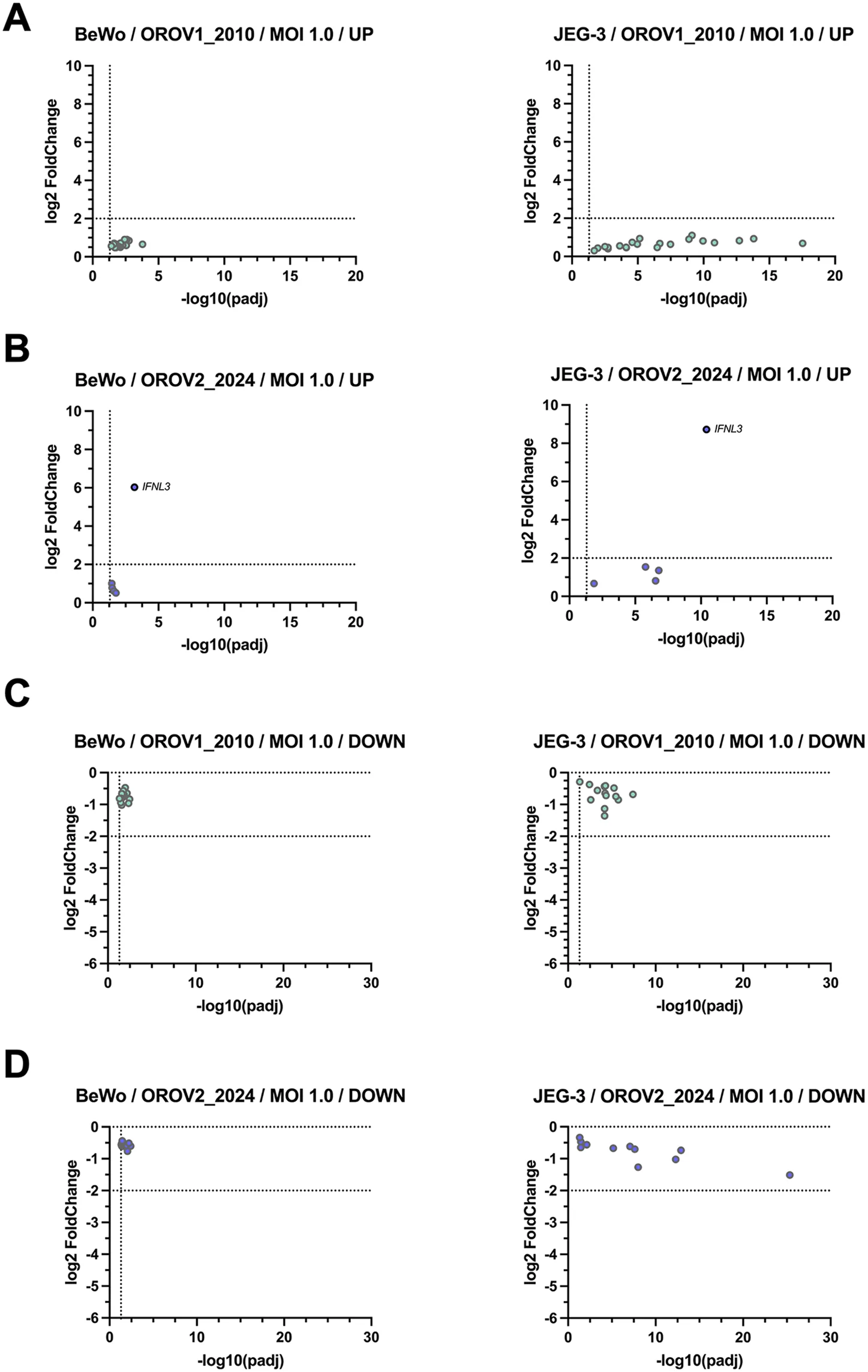
Transcripts common to infection of BeWo and JEG-3 infected cells. Transcripts from either OROV1_2010 (green) or OROV2_2024 (purple) infection and were common to infection of BeWo (MOI 1.0) and JEG-3 (MOI 1.0) cells were identified. Biological significance was defined as log2 foldchange >2 or <-2 (y axis) and adjusted p value <0.05 (x axis). (A, B) Transcripts that increased in OROV1_2010 or OROV2_2024 infection, respectively. (C, D) Transcripts that decreased in OROV1_2010 or OROV2_2024 infection, respectively.

To investigate whether JEG-3 and BeWo cells respond similarly to OROV infection, we next performed pathway analysis on common DEGs in these two trophoblast cell lines. Infection in MOI 0.1 cells was omitted due to the very small number of differentially expressed transcripts in BeWo cells identified above (Fig 3). We found a much smaller number of transcripts were common to either OROV1_2010 or OROV2_2024 infection (S9 Table). The identity of these transcripts was different in OROV1_2010 or OROV2_2024 infected cells, indicating differential virus-host interactions of both OROV1_2010 and OROV2_2024. In OROV1_2010 infected cells, there were 21 common transcripts upregulated, but none of these reached the biologically significant log2FoldChange of 2 (Fig 9A). OROV2_2024 infected cells had only 5 common transcripts and only one was above the log2FoldChange of 2 this was the known antiviral factor interferon lambda 3 (*IFNL3, or IFNλ3*) (Fig 9B). In the downregulated transcripts OROV1_2010 and OROV2_2024 infected cells had no common transcripts that reached our biologically significant threshold of -2 (Fig 9C-9D).

**Fig 10:**
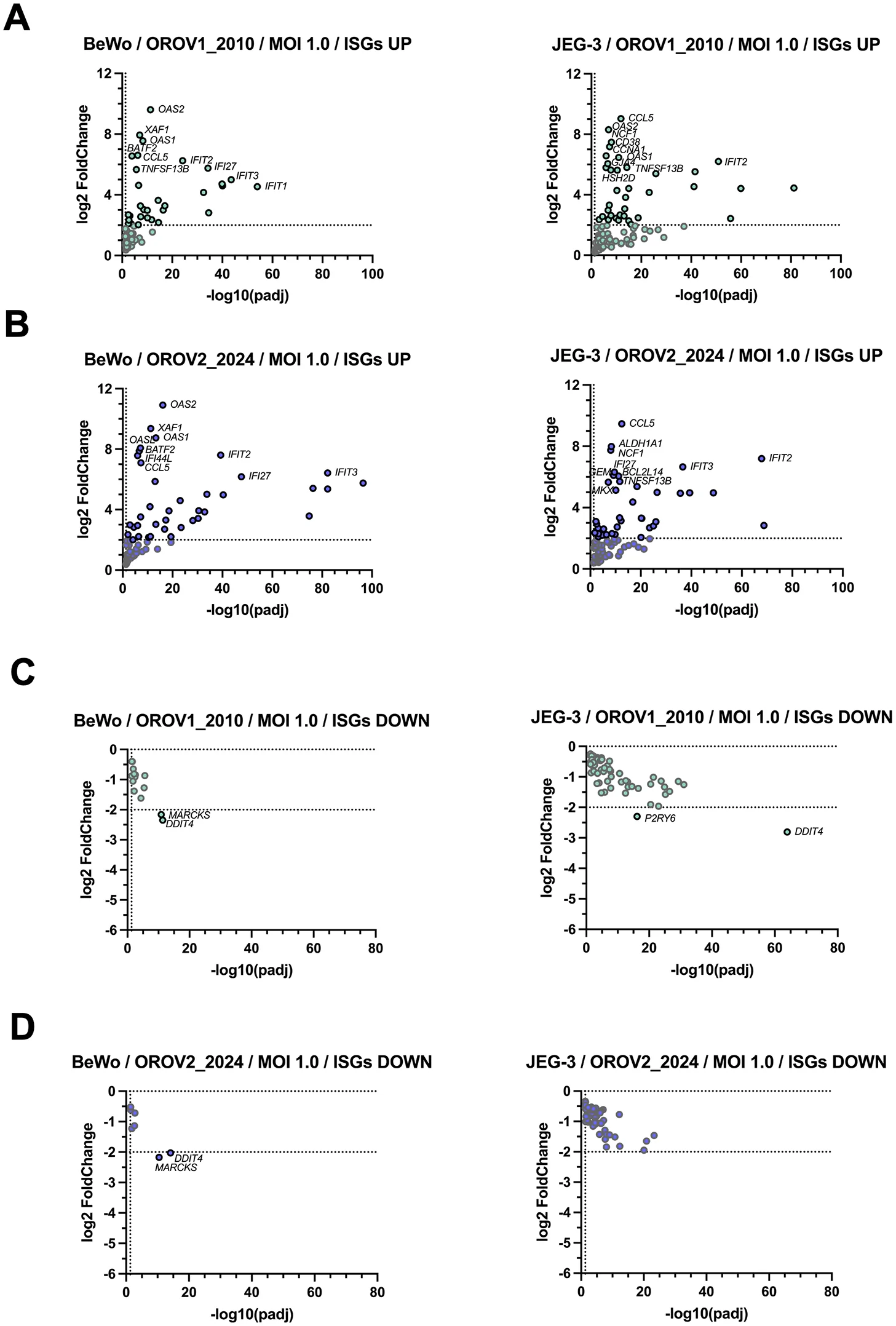
Expression of ISGs in OROV1_2010 and OROV2_2024 infected cells. All transcripts that increased or decreased upon OROV1_2010 (green) or OROV2_2024 (purple) infection in BeWo (MOI 1.0) and JEG-3 (MOI 1.0) cells were analyzed for the presence of ISG transcripts. Biological significance defined as log2 foldchange >2 or <-2 (y axis) and adjusted p value <0.05 (x axis). (A, B) Transcripts that increased in OROV1_2010 or OROV2_2024 infection, respectively. Ten transcripts with largest log2FoldChange labelled. (C, D) Transcripts that decreased in OROV1_2010 or OROV2_2024 infection, respectively.

**Fig 11:**
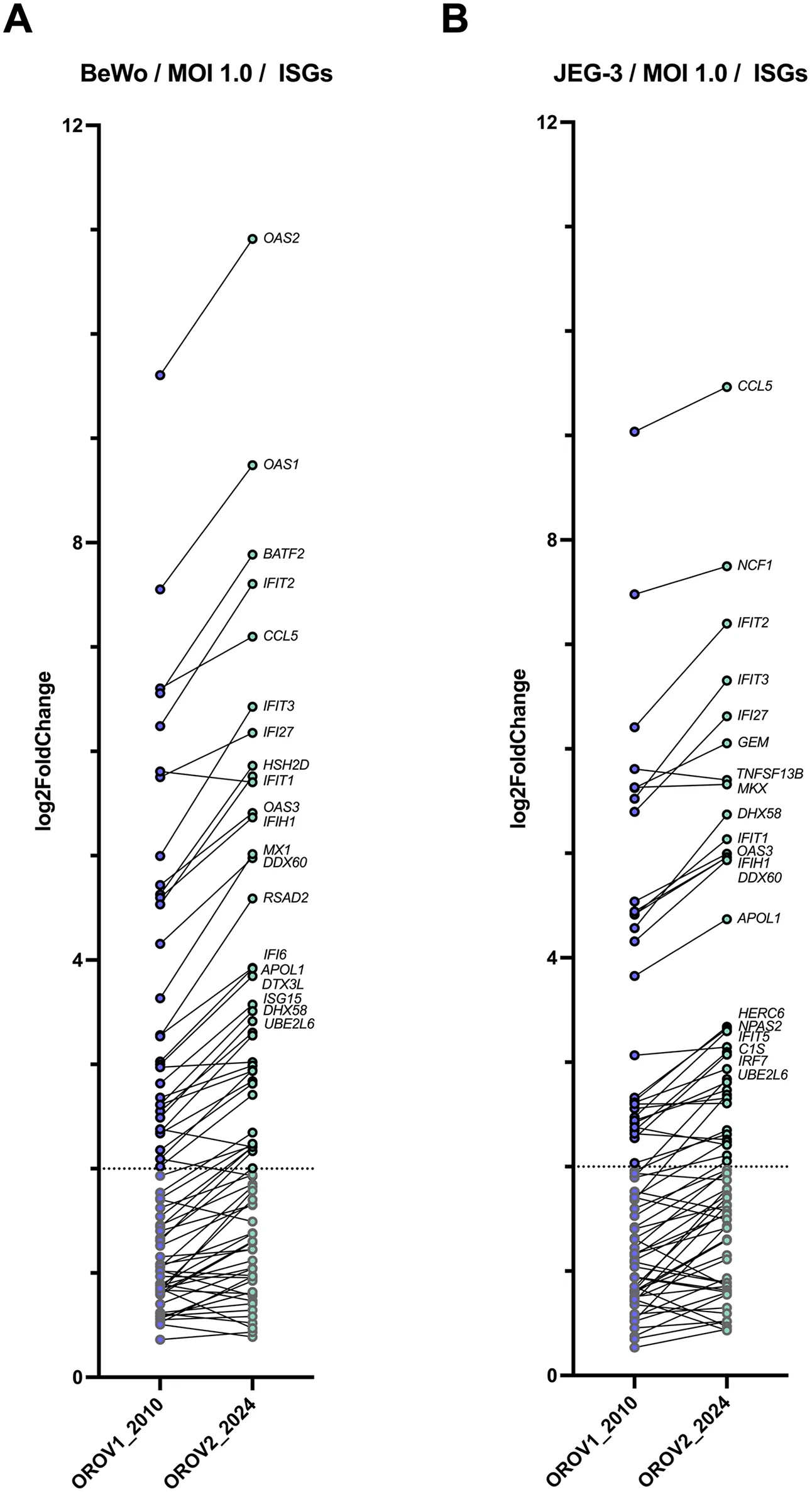
Comparison of Log2FoldChange of ISG expression in OROV1_2010 and OROV2_2024 infected cells. Expression of transcripts of ISGs differentially expressed in OROV1_2010 (green) or OROV2_2024 (purple) infection in BeWo (MOI 1.0) **(A)** and JEG-3 (MOI 1.0) **(B)** were compared. Biological significance was defined as log2 foldchange, with a biological significance cut of set at >2. Twenty transcripts with largest log2FoldChange are labelled.

As IFNL3 is a known antiviral factor that stimulates the transcription of a range of other antiviral factors [39], we then analysed if expression of known interferon stimulated genes (ISGs) increased or decreased in either OROV1_2010 or OROV2_2024 infected cells. We compared a curated list of known interferon stimulated genes (ISGs) [40] with those transcripts that increased or decreased during OROV1_2010 or OROV2_2024 infection (Fig 10 and S10 Table). Overall, in BeWo cells a nearly identical number of ISGs had upregulated expression with 69 in OROV1_2010- and 72 OROV2_2024-infected cells, conversely there was a difference in downregulated ISG expression during infection with 14 in OROV1_2010- and 7 in OROV2_2024-infected cells. In contrast, this difference appeared reversed in JEG-3 cells whereby the upregulated ISGs were different between strains- with 101 in OROV1_2010- and only 84 in OROV2_2024-infected cells; but downregulated ISGs were similar with 59 and 56, respectively (S10 Table). The repertoire of interferon stimulated genes expressed in OROV1_2010 and OROV2_2024 was nearly identical and there were only two ISGs uniquely expressed in OROV2_2024 compared to OROV1_2010 infected cells that were common in both BeWo and JEG-3 cells, *APOL6* and *IL6ST* (S10 Table). However, it was notable that the magnitude of gene expression change (log2FoldChange) of nearly every interferon gene identified was greater in OROV2_2024 infected cells compared to OROV1_2010 infected cells in both BeWo and JEG-3 cells (Fig 11). Therefore, we conclude that *IFNL3* expression in OROV2_2024 infected cells, compared to OROV1_2010 infected cells (Fig 10), is associated with an increase in expression in a very broad range of ISGs in OROV2_2024 infected cells, compared to OROV1_2010 infected cells (Fig 11). This difference in expression of interferon stimulated genes may be reflected in the difference in OROV plaque sizes that we observed above.

## Discussion

Here we conducted a comparative study of an ancestral and recent isolate of OROV to assess potential differences related to host responses in cell lines derived from placenta. This is critical to understanding any potential differences that may be linked to effects on fetal development. Although many virus-host interactions in the placenta remain very poorly characterized, there is a growing understanding of the innate immune response to infection in placental cells. The innate immune responses of differing trophoblast cultures to diverse DNA and RNA viruses are distinct from one another [41], and there is increasing evidence that type III interferons, such as the interferon lambda proteins, are required to control RNA virus replication in the placenta [42]. Perhaps the most advanced example of this is the evidence demonstrating the importance of IFNL proteins, including IFNL1 and IFNL3, in protecting against Zika virus (ZIKV) infection in placental tissues [43–45]. Infection of BeWo and JEG-3 by OROV has been previously shown [28,46], also the induction of type I interferon responses in these cells when infected OROV BeAn19991 (a prototype strain) and control of transmission to the fetus in a murine model [46]. This study adds a comparative angle showing differences to a recent isolate, and by using a selected number of genes the authors show induction of type I interferon responses (with minor differences) to infection in both cell lines. Our study goes further in that we provide an in-depth analysis of full transcriptome rather than selected genes, suggesting differences between responses depending between cell lines and virus -for example related to apoptotic processes, protein transport- as well as common aspects such as innate immune and antiviral responses. Importantly, we describe that the amplitude of innate immune response is stronger with OROV2_2024 compared to OROV1_2010 in both cell lines with a degree of overlap in ISG expression, pointing potentially to a common virus-dependent factor that may antagonise host responses (also see below) as well as (to some degree) comparable cellular detection and response mechanism(s) to OROV. Our observation that OROV2_2024 infection, which induces a small plaque phenotype BeWo and JEG-3 cells and induces *IFNL3* is interesting; indeed, the previously mentioned study [46] also described induction of *IFNL* in JEG-3 cells. At present the functional relevance of IFNL3 in this context remains unclear. It is worth mentioning that the type III interferon IFNL1 been described to protect both trophoblast and non-trophoblast cells from ZIKV infection [43], thus this observation may be relevant. It would require *IFNL3* KOs or neutralisation assays for example to assess its contribution to restricting OROV2_2024. In time, further work may shed light on this observation and add to the growing literature indicating that IFNL proteins, including IFNL3, are important for controlling infection of placenta cells by diverse RNA viruses. Also, as there is functional redundancy between IFNL3 proteins [39], it is possible that IFNL3 is important in restricting OROV2_2024 replication in trophoblast cells, though other IFNL proteins may be expressed in other placental cells to restrict OROV infection *in vivo*. Furthermore, our observation that OROV2_2024 displays a small plaque phenotype in A549 cells suggests that restriction of replication may extend beyond placental cells. However, this point is speculative and requires further examination. As discussed, whether the small plaque phenotype is due to viral cell-to-cell spread, viral replication, virion release etc. or a combination of processes is not clear and may require further investigations. Our present data do not allow us to come to conclude on these points. It is however worth noting that this difference is also seen in A549-IRF3-KO cells and thus is unlikely to be dependent of a full type I interferon response; intrinsic factors of either cellular or viral nature, or both, might underpin our observations and this will require further studies. Indeed, it remains unknown what factors are required for the sensing of OROV2_2024 in the placental cells we have used here. However, data from a murine model using the prototype OROV strain BeAn19991 indicates that OROV can be sensed by a RIG-I-like pathway involving the mitochondrial antiviral signalling (MAVS) protein [47] and it is possible this pathway is active in our experiments. It is thus interesting to note the difference in *IFNL3* induction between OROV1_2010 and OROV2_2024 infected cells, though as stated above the functional consequences do require further investigation. This may suggest that a viral factor involved in antagonism of IFN production differs between the two OROV isolates used here. It has been reported that the NSs protein from OROV BeAn19991 can antagonize type I interferon production and requires the function provided by the extreme carboxyl terminus if the NSs protein [48]. This also requires further exploration, as differences in NSs sequences or expression may be linked to differences in inhibition of interferon induction. Indeed, there are some noticeable changes in NSs proteins of OROV1_2010 compared OROV2_2024 and AM0088, the latter being almost identical (S1 Fig). The functional relevance remains to be determined, though reverse genetics and type I IFN reporter or production assays offer possibilities to assess if recent isolates may have an NSs protein that is a weaker antagonist of host responses. Future studies may investigate these aspects. It can however not be ruled out that other viral factors are at play, or host responses that not directly IRF-3 dependent. This study did not elucidate those points but opens the door to such studies.

Our data suggest that expression of the interferon stimulated genes is associated with expression of IFNL3. However, it remains unknown if one or many of these proteins interfere with OROV. Indeed, future studies may also want to look at the dynamics of immune response induction in details as these processes develop over the course of infection, and further differences between these viruses may be observed. It is unknown what ISG proteins restrict any OROV virus, with the exception of ISG20 [49]. It has however been demonstrated that specific ISG proteins, such as IFITM3, IRF1, HPSE and ISG20, can restrict replication of other bunyaviruses including those related to OROV [49,50]. However, it is possible that differences in ISG protein action vary between bunyaviruses and/ or that a combination of the wide range of ISGs we find expressed in OROV cells are responsible for restricting OROV replication, potentially in an isolate dependent manner. Any ISGs limiting OROV2_2024 replication in the presence of IFNL3 might be affecting cell to cell spread of the virus, but not its release into the extracellular milieu. It is in this context interesting to consider if potentially reduced pathogenicity may in fact contribute to fetal infection. Indeed, for ZIKV it has been suggested that African isolates have higher pathogenicity than Asian ones, resulting in fetal death rather than malformations [51]. We have considered a similar question elsewhere, observing that the replication of the TORCH [52] pathogen human cytomegalovirus (HCMV) is limited in a trophoblast cell line (SGHPL-4 cells) [53]. In this case, we reasoned that limited virus replication of HCMV in trophoblast cells was advantageous to vertical transmission of HCMV across the placenta, as this would allow HCMV replication without profound tissue damage that would lead to miscarriage and inhibit vertical transmission of HCMV. It is possible that the limited ability of OROV2_2024 to spread from cell to cell in plaques allows an analogous situation, where OROV2_2024 can replicate in placenta cells without risking profound tissue damage that would cause miscarriage, but still transfer to the fetus. We propose that IFNL3 stimulated antiviral protein expression directly or indirectly affects OROV2_2024 replication in placental cells. It is interesting to speculate that increased control and/or lower pathogenicity of recent OROV isolates may contribute to what is observed with other viruses including ZIKV. However, this requires further investigations and effects on fetuses observed may just be due to higher case numbers and vigilance of public health responses. Indeed, the cell culture systems used in this study cannot fully represent the complexity of the placenta with its complex physiological functions, including as protective barrier [54]. More complex systems for *in vitro* and *ex vitro* investigations are available nowadays for more in-depth studies such as primary trophoblast cells, placental explants or organoids etc. all bring their own advantages and/or challenges that come with organisation, complexity, origin that may impact experiments and design [55] but are nonetheless worthwhile considering.

It is still too early to speculate on the usefulness of IFNL3 as an antiviral intervention against some OROV strains, and indeed related viruses. It has been previously demonstrated that IFNL3 robustly inhibits replication of influenza virus in murine models [56–58]. Importantly, the antiviral effects of IFNL3 were not associated with disadvantageous inflammatory side effects that are observed with using type I interferons [57,58]. Whether IFNL3 could be developed for use against OROV strains, even during pregnancy where activation of inflammation must be avoided, may indeed require the use of more sophisticated infection models to begin with including those mentioned earlier, e.g., placental explants, organoids etc. There are clearly limits in the use of cell lines used here but our analysis leads to testable hypotheses, and our findings may inform further studies on preventive measures on this and other pathogens.

## Supporting information

S1 Table Differential expression of genes in infected and uninfected BeWo cells (BeWo Differential Expression Results). (XLSX)

S2 Table List of statistically significant increased or decreased transcripts in infected BeWo cells (BeWo Significant Results). (XLSX)

S3 Table Differential expression of genes in infected and uninfected JEG-3 cells (JEG-3 Differential Expression Results). (XLSX)

S4 Table List of statistically significant increased or decreased transcripts in infected JEG-3 cells (JEG-3 Significant Results). (XLSX)

S5 Table List of statistically significant increased or decreased transcripts common or unique to either OROV1_2010 or OROV2_2024 in BeWo cells (BeWo VD Output). (XLSX)

S6 Table List of statistically significant increased or decreased transcripts common or unique to either OROV1_2010 or OROV2_2024 in JEG-3 cells (JEG-3 VD Output). (XLSX)

S7 Table Gene ontology of statistically significant increased or decreased transcripts common or unique to either OROV1_2010 or OROV2_2024 in BeWo cells (BeWo Gene Ontology). (XLSX)

S8 Table Gene ontology of statistically significant increased or decreased transcripts common or unique to either OROV1_2010 or OROV2_2024 in JEG-3 cells (JEG-3 Gene Ontology). (XLSX)

S9 Table List of differentially expressed genes common to BeWo and JEG-3 infection. (XLSX)

S10 Table Interferon stimulated genes expressed in OROV infection. (XLSX)

S1 Fig Amino acid differences between open reading frames of OROV1_2010 and OROV2_2024, compared to AM0088. (DOCX)
