# Supplementary material for: Comparative analysis of host responses to Oropouche viruses in placental cell infection models
Source: PLoS Negl Trop Dis. 2026 Sep 21;20(9):e0014593. doi: 10.1371/journal.pntd.0014593 (PMC13592696; doi:10.1371/journal.pntd.0014593)
Supplement: Supplementary file 1 [file pntd.0014593.s011.docx]

**S1 Fig. Amino acid differences between open reading frames of OROV1_2010 and OROV2_2024, compared to AM0088.** Changes shown where applicable, eg., for L (RNA-dependent RNA polymerase), M and NSs.
